# Structural evolution and strength change of a metallic glass at different temperatures

**DOI:** 10.1038/srep30876

**Published:** 2016-08-03

**Authors:** X. Tong, G. Wang, Z. H. Stachurski, J. Bednarčík, N. Mattern, Q. J. Zhai, J. Eckert

**Affiliations:** 1 Laboratory for Microstructures, Institute of Materials, Shanghai University, Shanghai 200444, China; 2 Research School of Engineering, CECS, Australian National University, Canberra ACT0200, Australia; 3 HASYLAB at DESY, Notkestr. 85, D-22603 Hamburg, Germany; 4 IFW Dresden, Institute for Complex Materials, Helmholtzstr. 20, D-01069 Dresden, Germany; 5 Erich Schmid Institute of Materials Science, Austrian Academy of Sciences, Jahnstraβe 12, A-8700 Leoben, Austria; 6 Department Materials Physics, Montanuniversität Leoben, Jahnstraβe 12, A-8700 Leoben, Austria

## Abstract

The structural evolution of a Zr_64.13_Cu_15.75_Ni_10.12_Al_10_ metallic glass is investigated *in-situ* by high-energy synchrotron X-ray radiation upon heating up to crystallization. The structural rearrangements on the atomic scale during the heating process are analysed as a function of temperature, focusing on shift of the peaks of the structure factor in reciprocal space and the pair distribution function and radial distribution function in real space which are correlated with atomic rearrangements and progressing nanocrystallization. Thermal expansion and contraction of the coordination shells is measured and correlated with the bulk coefficient of thermal expansion. The characteristics of the microstructure and the yield strength of the metallic glass at high temperature are discussed aiming to elucidate the correlation between the atomic arrangement and the mechanical properties.

By virtue of the atomic structure characteristic in metallic glasses, i.e., (i) no long-range periodic order, and (ii) an amount of order at medium- and short-ranges, excellent and unique physical, mechanical and chemical properties are found in these materials, which have attracted considerable interest over the years^1^. Copper mould suction casting is a widely applied method to obtain a variety of metallic glasses by quenching from the liquid state. During glass formation, defects are formed in the inhomogeneous structure, defined as deviations from ideal amorphous packing^2^. The dense random packing hard-sphere model is also known to describe the atomic structure of metallic glasses^3^. The defects in crystalline materials formed during solidification, such as vacancies, interstitial atoms and dislocations, are the key factors that determine the material performance^1^. It is conjectured that the defects in the amorphous atomic arrangement also play a crucial role in determining the performance of metallic glasses. The deformation and fracture mechanisms of metallic glasses are fundamentally different from those in crystalline solids^1^. In particular, the deformation and fracture mechanisms are associated with the inhomogeneous structure at the nanoscale^1^. The atomic structure and possible defects at nanoscale are difficult to be observed in the complex and disordered structure, even when applying high-resolution transmission electron microscopy (HRTEM). Dynamic relaxation is also a phenomenon to reflect the inhomogeneous structure indirectly^4^.

It is well known that metallic glasses are thermodynamically unstable or metastable, with excess Gibbs free energy, which means that crystallization will begin if sufficient energy is supplied to the glassy solid. For example, in metallic glasses used for the outer wall shield on the spacecrafts^5^,^6^,^7^, the temperature of the shield can reach more than 500 K. As a result, the metallic glass may experience a structural evolution. Then, the protection ability of the outer wall shield may be different from that at room temperature when it is tested by a hypervelocity impact. Furthermore, under a uniaxial applied force, the structure of the amorphous phase becomes anisotropic^8^,^9^. For these and many other reasons it is necessary to investigate the temperature dependence of the structure evolution. *In-situ* high-energy synchrotron X-ray radiation is one technique that can provide sufficient energy and resolution for exploring the atomic structure evolution with temperature during heating. Recent published work revealed diverse structural changes occurring upon loading or heating using *in-situ* high-energy synchrotron X-ray radiation^8^,^10^,^11^,^12^,^13^,^14^,^15^.

Under an external stress, a concordantly local shifting of atoms will occur^16^, which can bridge the macroscopic yield strength and the microscopic atomic structure^14^. Upon heating, the thermal expansion and the changes in free volume can be measured^17^,^18^,^19^. The increasing free volume is an integral part of the deformation process, which is accumulated with deformation strains^20^.

The purpose of this work is to unravel the structural evolution in a Zr_64.13_Cu_15.75_Ni_10.12_Al_10_ metallic glass with temperature *in-situ* during heating from room temperature up to crystallization using high-energy synchrotron X-ray radiation. The temperature dependence of the structure in reciprocal space and in real space is characterized. The shear transformation is used to elaborate the deformation process of the metallic glass at different temperatures.

## Structural evolution in reciprocal space

**Figure 1:**
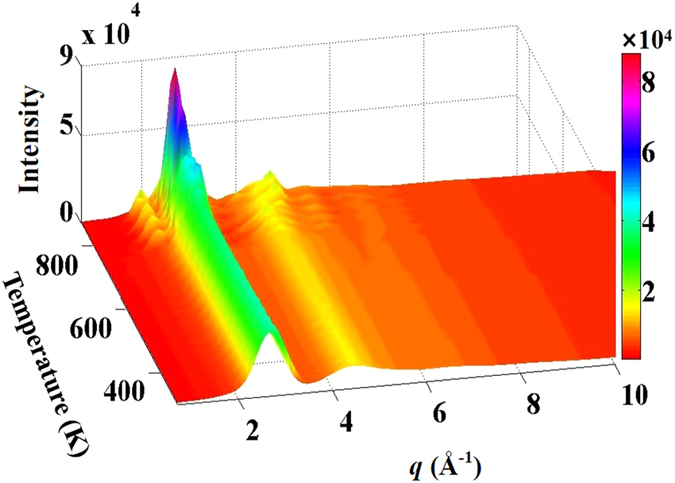
Series of *in-situ* high-energy synchrotron X-ray radiation patterns from room temperature up to 870 K (crystallized temperature) in reciprocal space, *q* = 2*π* sin *θ*/λ.

**Figure 2:**
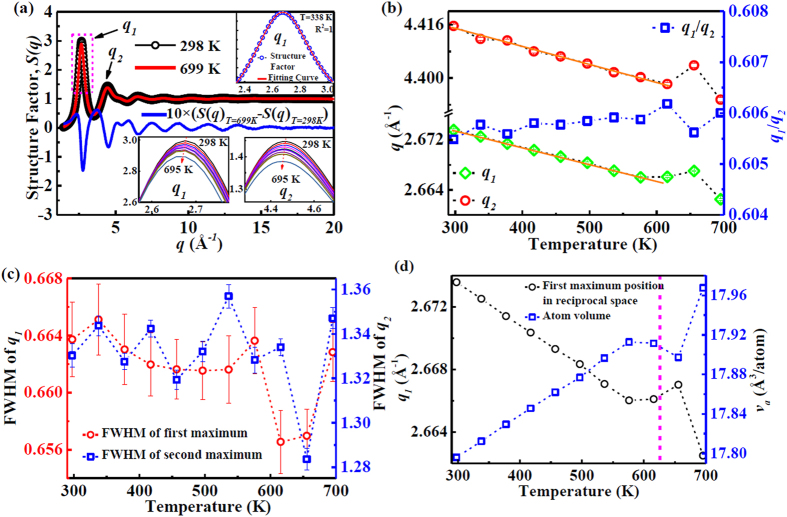
Structure evolution in reciprocal space. (**a**) The structure factors of full amorphous (at 298 K) and crystallized (at 699 K). The right inset shows the first peak in structure factors which was fitted by a *pseudo-Voigt* function. Insets at left and at right show the enlarged first maximum and the enlarged second maximum, respectively. (**b**) The positions of the first (*q*_*1*_) and second (*q*_*2*_) maxima in the structure factors ranging from 298 K to 699 K. The orange lines show the linear fitting of the positions which was interrupted near 616 K. (**c**) Full width at half maximum (FWHM) of the first and second maxima in the structure factors as a function of temperature. (**d**) The first maximum position in the structure factor and the average volume of the metallic glass sample calculated from the relation *q*_*1*_ ∙ *v*_a_^0.433^ = 9.3.

The high-energy synchrotron X-ray results provide diffraction intensity curves, *I*(*q*), as shown in Fig. 1. These curves reveal the evolution of the crystallisation process. The *I*(*q*) curves at low temperatures, ranging from 298 K to 699 K, show the glassy nature of the specimens. When the temperature is higher than 699 K, the metallic glass gradually devitrifies from the fully glassy structure, and the intensity of the first maximum in the *I*(*q*) curve becomes sharper. The structure factor, *S*(*q*), of the metallic glass at 298 K and 699 K is plotted in Fig. 2(a). The difference curve of Δ*S*(*q*) ( = *S*(*q*)_*T*=699 K_ − *S*(*q*)_*T*=298 K_), multiplied by a factor of 10, is also given in Fig. 2(a). Due to the asymmetric contributions of Δ*S*(*q*) on the low *q* side, the peak positions shift to a lower *q* value, as shown by the insets below left for the peak at *q*_*1*_ and right for the peak at *q*_*2*_. To further quantitatively explore the structure information hidden in *S*(*q*), the first and second maxima are fitted by a *pseudo-Voigt* function that can accurately describe the profile including the intensity and the position of the maxima, and the full width at half maximum (FWHM) evolving with temperature^21^,^22^. The fitting process of the first maximum at 338 K is shown in the top right inset of Fig. 2(a). The positions of the first and the second maxima in *S*(*q*), and the FWHM of the first maximum are plotted in Fig. 2(b,c), respectively, in the temperature range from 298 K to 699 K. In the temperature range higher than 700 K, the crystalline phase precipitation and growth cause an asymmetric distribution in the maxima of *S*(*q*) (not shown). From Fig. 2(b,c) it can be seen that in the temperature range from 298 K to 616 K the positions of the first and second maxima linearly shift to a low *q* value with increasing temperature. Further increase in temperature causes the positions of the first and second maxima to change non-monotonously, i.e., the position shifts to a higher *q* value from 616 K to 656 K. After that, the positions of the maxima shift to the low *q* value. The FWHMs of the first and second maxima are plotted as functions of temperature, showing a non-monotonic change. It has been reported that the position of the first maximum follows a power-law scaling relationship versus atomic volume, *v*_a_^23^:


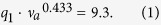


Accordingly, the variations of atomic volume and the position of the first maximum as functions of temperature are drawn in Fig. 2(d). Below 616 K (which is close to the glass transition temperature of 625 K), the atomic volume and the position of the first maximum behave apparently linearly with temperature. Above 625 K up to 656 K, the *q*_*1*_ value deviates from the linear relationship, and then decreases rapidly above 656 K. The *v*_a_ also correspondingly shows similar change. Clearly, in the temperature range from 625 K to 656 K some structural changes occur which involve rearrangements at the atomic level.

**Figure 3:**
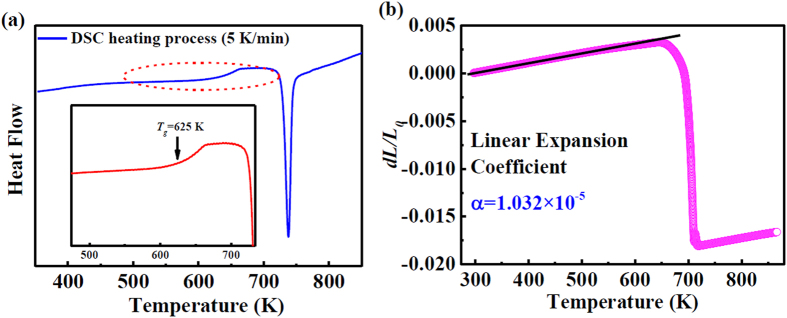
Thermal behaviours of the metallic glass. (**a**) Differential scanning calorimetry (DSC) scan measured upon heating from 300 K up to 863 K. The inset shows an enlarged picture of region marked by the red short dash circle. (**b**) The curve of *dL/L*_*0*_, measured by dilatometer, versus temperature drawn in (b) from which the linear expansion coefficient can be calculated as *α* = 1.032 × 10^−5^ K^−1^.

A typical DSC trace is shown in Fig. 3(a) to further characterize the structural evolution. The inset shows an enlarged region indicated by the red short dash circle, revealing the calorimetric glass transition temperature, *T*_*g*_, at approximately 625 K and the crystallization temperature, *T*_*x*_, at approximately 719 K. The onset of the drastic changes near 625 K observed at the positions of the first and second maxima are caused by local topological fluctuations, that is, the dynamics of the supercooled liquid become more heterogeneous compared with the static structural order^24^,^25^. Another measured property is the coefficient of thermal expansion shown in Fig. 3(b) which will be discussed later.

## Structural evolution in real space

### a) Thermal expansion and contraction

**Figure 4:**
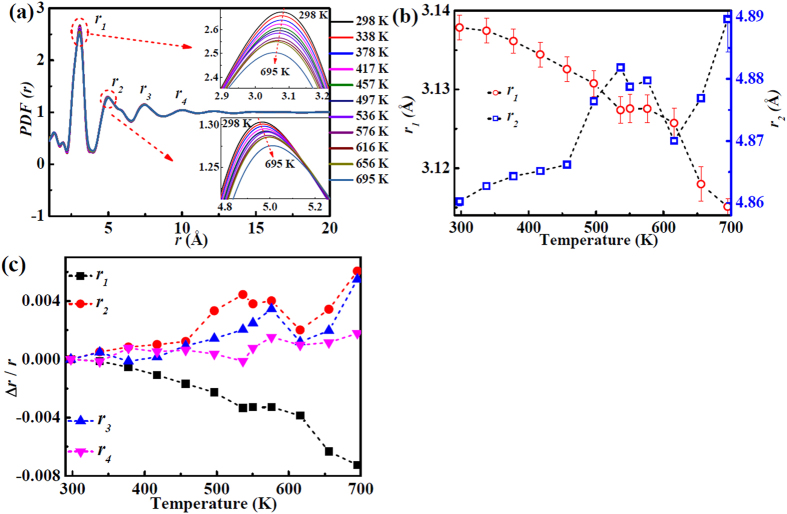
Structure evolution in real space. (**a**) The pair distribution function (*PDF*) from 298 K to 695 K. An inverse trend is shown for the enlarged first and second maxima in the insets. (**b**) The position values of the first maximum (*r*_*1*_) and the second maximum (*r*_*2*_) versus temperature, which result from the fitted *pseudo-Voigt* function. (**c**) The rates from the first to the forth maxima positions changing with temperature from 298 K to 616 K are calculated by Δ*r*/*r*.

To probe the atomic structure evolution with temperature in real space, a noteworthy structural information is shown in Fig. 4(a). The pair distribution function (*PDF*) reveals that the positions of the first maximum (*r*_*1*_), and the second maximum (*r*_*2*_) change with temperature. Enlarging the maxima in *PDF* shows that the first maximum position shifts to lower *r* values, and that the second maximum position shifts to higher *r* values when the temperature increases from 298 K to 695 K. The peak positions corresponding to *r*_*1*_ and *r*_*2*_ can also be fitted by a *pseudo-Voigt* function^22^. The changing values versus temperature are shown in Fig. 4(b). The *r*_*2*_ values shift to high *r* values, increasing slowly before 457 K, followed by larger changes between 457 K and 616 K, and increasing rapidly again after 616 K. By the same method, the positions of the third, fourth, and fifth maxima were also fitted (not shown here). The rates of the changing positions with temperature, calculated as Δ*r*/*r*, are shown in Fig. 4(c). The relative change of the first maximum decreases with increasing temperature. The relative changes of second and third maximum remain relatively constant before 457 K, then increase obviously in the temperature range of 457 K to 536 K, followed by a decrease before *T*_*g*_ and an increase after *T*_*g*_. The forth shell stays almost constantly before 536 K, and expands above 536 K. The thermal expansion coefficients of the different shells have been calculated from Fig. 4(c) and are listed in Table 1. The temperature range for shell-expansion calculation is from 298 K to 616 K to avoid the influence of the glass transition. The coefficients can be compared and are found to deviate from the macroscopic thermal expansion [see Fig. 3(b)]. However, the total sum of the individual shell coefficients shown in Table 1 is within the experimental error of the measured macroscopic bulk coefficient as given in Fig. 3(b). The value of the linear expansion coefficient, *α*, is 1.032 × 10^−5^ K^−1^. The thermal expansion coefficient shows a sudden drop around 650 K due to the transition of material from the glassy state to the supercooled liquid state^26^, and deviates from the linear change from 625 K.

### b) *RDF* peak position by atomic pairs

**Figure 5:**
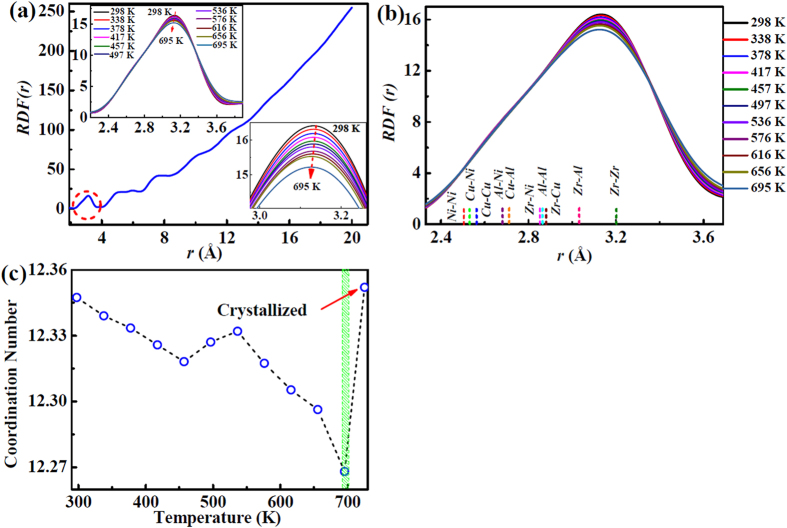
Structural information in *RDF*. (**a**) Radial distribution function, *RDF*(*r*). Insets show the enlarged first shell in *RDF*(*r*), shifting left from room temperature to high temperature; (**b**) The possible atomic pairs in the first shell ranging from 298 K to 695 K; (**c**) The coordination numbers, changing with temperature.

To explore the underlying relationship between the evolution of atomic structure and temperature, the radial distribution functions (*RDFs*) at different temperatures are plotted in Fig. 5. The enlarged first maximum of *RDF*(*r*) in the left inset of Fig. 5(a) shows a leftwards shift with increasing temperature. Figure 5(b) shows the positions of all partial pair correlations that make sub-peak contributions to the first maximum. The positions of the sub-peaks for each pair are calculated on the basis of the theoretical bond lengths of the component elements (*R*_*Zr*_ = 1.60 Å, *R*_*Cu*_ = 1.28 Å, *R*_*Ni*_ = 1.25 Å, and *R*_*Al*_ = 1.43 Å), as listed in Table 2.

In the fully glassy state, the atomic pairs are composed of small atoms, such as Cu-Cu, Cu-Ni, Ni-Ni, medium sized atomic pairs, such as Zr-Cu, Zr-Ni, Cu-Al, and big atomic pairs, Zr-Zr. The probability of these pairs of atoms can be calculated by the random contact formula, 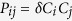 (*C*_*i*_ and *C*_*j*_ are the atomic concentration of elements *i* and *j*, respectively, and *δ* = 1 when *i* = *j*, or *δ* = 2 when *i* ≠ *j*). These probabilities are listed in Table 2. As the content of Zr element is high, the Zr-Zr pairs dominate the atomic packing.

The coordination numbers versus temperature of the first shell are shown in Fig. 5(c), calculated by integration of the first maximum^27^, revealing that the average coordination number in the first shell remains approximately constant as 12.3 ± 0.02 up to near 700 K.

### c) Exchange of atoms

The apparent thermal contraction or expansion of the coordination shells is the result of at least two effects: (i) normal thermal expansion due to anharmonic thermal vibrations, and (ii) exchanges of atoms of different sizes. The atoms in the first nearest-neighbour shell undergo thermal vibrations to make a self-coordination during heating^28^. We conjecture that during heating from room temperature an exchange of atoms takes place between the first and the second coordination shells by an atomic concordant displacement^10^,^14^,^29^. In particular, a Zr atom moves from shell one to shell two, in exchange for one of the small atoms (Cu or Ni) jumping into shell one. It is known that the Zr atoms can change site with Cu or Ni atoms^30^ and that the Zr-Cu interatomic distance appears not to change with increasing temperature^31^. This has the effect of reducing the effective diameter of the first shell, and reducing the concentration of Zr atoms around the central Zr atom. Consequently, the number of small atomic pairs, and big atomic pairs decreases during the heating process. The atomic pairs of medium interatomic distance, like Zr-Cu, Zr-Ni, Zr-Al, will come into being.

These atomic exchanges between the first and second coordination shells overcome the normal thermal expansion, and cause the negative thermal expansion observed in the first shell of *PDF* [see Fig. 4(b)]. Since the results in Fig. 4(b) are determined from X-ray scattering, the occurrence of Zr-Zr and other atomic pairs is made more significant by their scattering power, and therefore one must use the weighting factors, *W*_*ij*_, of the atomic pairs, calculated by 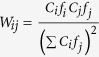 (where *C* is the atomic number of element; *i* and *j* represent different elements; *f* is the atomic fraction of element)^27^, listed in Table 2. From the *W*_*ij*_, the dominant atomic pairs are mainly Zr-Zr, Zr-Cu, Zr-Ni, and to a small degree Zr-Al, which contribute to the first coordination shell. The weighting factors of the other six pairs have small occupations and relatively weak scattering powers because of their low concentrations^13^.

### d) Frozen-in stresses

On rapidly quenching the liquid solidifies with close to random atomic arrangement, as would be predicted by statistical probability^2^. Such an arrangement would be stable if there were no differences in the interatomic forces between the four elements. However, there are strong thermodynamic preferences for all of the possible 10 pairs^13^,^30^. Therefore, the initial quenched atomic structure is expected to be far from equilibrium and contains frozen-in stresses. The measured thermal contraction and expansion of concentric coordination shells is a remarkable phenomenon discovered only in metallic glasses. These changes are irreversible (i.e., different from the anharmonic deformations) and therefore can be ascribed to the frozen-in stresses during solidification and their subsequent relaxation upon heating. The negative and positive changes, caused by the frozen-in stresses, must satisfy the principle of mechanical equilibrium: Σ *F* = 0, where *F* represents in this case an element of spherical surfaces, formed by the respective coordination shells. Due to spherical symmetry the total surface force acting at *r*_*1*_ = 3.138 Å (the first peak position in *PDF* at 298 K) is calculated from: 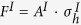, 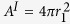, *σ*_*h*_ = *K*(Δ*V/V*). The change of volume is calculated as 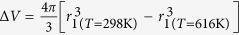. The bulk modulus, *K*, of this metallic glass is 106.6 GPa^32^. Therefore, *F*^*I*^ ≈ −1.52 × 10^−9^ N. A similar calculation for the second coordination shell is complicated by the choice of the radius of the shell. If the position of the second peak is chosen as the radius, *r*_*2*_ = 4.86 Å (the second peak position in *PDF* at 298 K), then the calculation gives the value: *F*^*II*^ ≈ 1.92 × 10^−9^ N. The imbalance of the surface forces between the first and second coordination shell is accommodated as the magnitude of the frozen-in stresses.

## Structural evolution in the supercooled liquid state

### a) Intermediate temperature stage

Before the crystalline stage is reached (870 K), the atomic arrangement must undergo sufficient structural rearrangement to allow formation of nuclei and crystal growth. We envisage this to happen through superposition of two collocated processes:

1. Increasing local density contrast (modulation) by the atomic exchanges described above in relation to coordination shells, and
2. sporadic formation of nuclei through the icosahedral cluster formation.

**Figure 6:**
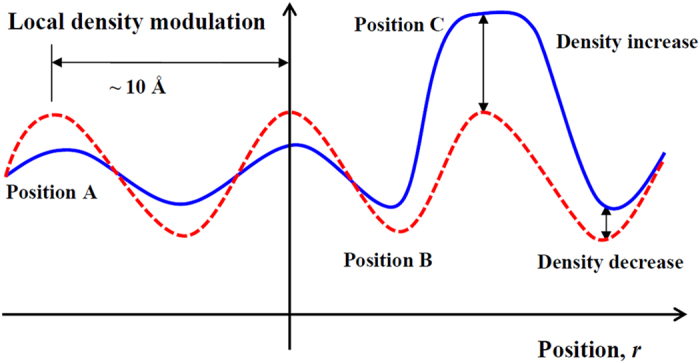
Schematic representation of local density variation in the metallic glass. Red dash line — initial density fluctuation resulting from random packing at high cooling rates. Blue line — Smoothed density modulation after annealing with occasional high density (Position C) due to formation of atomically ordered nucleus. The meaning spacing between local density variations can change with annealing of the metallic glass. The nearest average distance of the local atomic packing is about 10 Å^2^,^64^,^65^.

Random packing of spheres as a model of atomic arrangement in the metallic glass predicts variations in local coordination numbers and consequently variation in local density at the atomic scale^33^,^34^. We can schematically represent this variation as shown in Fig. 6, in which, positions A have higher density than positions B due to: (a) high coordination number, (b) possible short-range order (SRO) and medium-range order (MRO) which results from atomic rearrangements of random packing, driven by minimization of Gibbs free energy, and for this metallic glass, (c) segregation of (impurity) elements: Zr_64.13_Cu_15.75_Ni_10.12_Al_10_ → SRO + MRO + matrix of altered composition.

By contrast, positions B have lower relative density due to: (a) low coordination number of the cluster and therefore higher content of free volume, (b) lower atomic weight atoms due to rearrangement of atomic positions driven by frozen in stresses, and impurity segregation by enthalpic forces, and (c) for this metallic glass, changed composition inducing lower *T*_*g*_ compared to position A.

Positions A and B repeat throughout the volume of the glass with a periodicity characteristic of the alloy. By a random process, positions C are created at points of already high density, at which the local atomic composition and arrangement is conducive to formation of a stable ordered nucleus of near crystalline arrangement.

The icosahedral atomic arrangement offers an intermediate step between random atomic packing and fully crystalline packing. It has a density approaching that of the crystal, an internal interaction energy comparable to that of *fcc* structure, yet a surface energy more than an order of magnitude lower, thus allowing much smaller nuclei to be stable. The view, summarized by Spaepen^35^, is that the structure of supercooled liquids can be best described by packing of tetrahedra. The clusters undergo rapid atomic rearrangements towards a more ordered, crystalline structure. Such structures have been reported previously^36^,^37^,^38^. The nucleation and growth of nano-crystallites alters the modulated density landscape of the metallic glass represented initially by the field of positions of type A and B in Fig. 6. The fine scale of this modulation of the order of several nanometers coarsens to a much larger scale of tens of nanometers (the size of the nanocrystals ~50 nm). The final structure thus comprises (at 870 K) approximately 50% of nanocrystals embedded in a matrix of significantly changed composition.

### b) High temperature stage

**Figure 7:**
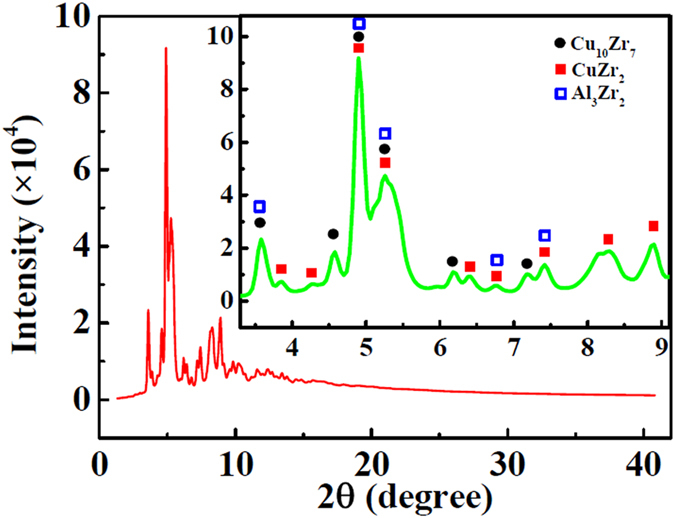
Synchrotron X-ray scattering curve at 870 K, showing the three crystalline phases.

In the four-element metallic glass studied here there is a possibility for many intermetallic compounds to form at specific locations where the initial packing at random can create favorable atomic arrangements. At 870 K, the thermodynamically stable crystalline phases (intermetallic compounds) selected from a large number of possible phases includes (in order of presence detected by X-ray scattering): CuZr_2_, Cu_10_Zr_7_, Al_3_Zr_2_ shown in Fig. 7 and possibly Ni_10_Zr_7_ not visible in Fig. 7.

**Figure 8:**
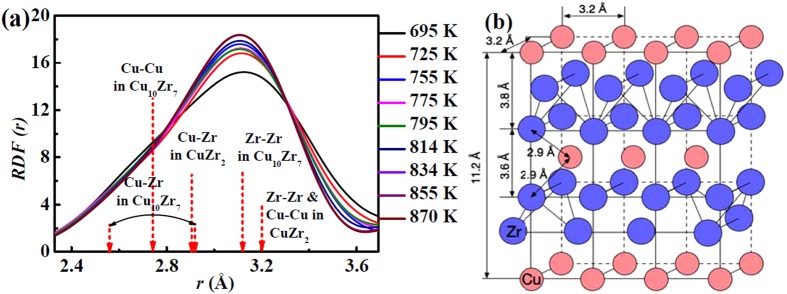
Atomic structure in the first nearest neighbour shell. (**a**) The atomic pairs of the last two compounds after crystallisation dominate in the first shell ranging from 695 K to 870 K in *RDF*. (**b**) The geometry of the crystalline CuZr_2_ cluster, indicating the interatomic distances.

There are at least 8 compounds that can form in a Cu-Zr binary system. However, of the four stable phases (Cu_5_Zr_8_, Cu_8_Zr_3_, Cu_10_Zr_7_, CuZr_2_), only the last two become predominant as the other two decompose into the other stable phases (CuZr → Cu_10_Zr_7_ + Cu_5_Zr_8_, and Cu_5_Zr_8_ → Cu_10_Zr_7_ + CuZr_2_)^39^,^40^ shown in Fig. 8(a). The pair distances of Zr-Zr, Cu-Cu and Cu-Zr in CuZr_2_ are obtained from Fig. 8(b), showing the nanocrystalline clusters^41^, and the pair distances of those in Cu_10_Zr_7_ are from ref. [40].

In the Ni-Zr binary system also 8 intermetallic compounds can form^42^. The formation of these at around 730 K is considered to be a process of short-range rearrangements in the amorphous structure. Lee *et al*.^43^, suggested that Ni_11_Zr_9_ and NiZr_2_ are the most stable of the possible 8 crystalline phases. However, it was suggested by ref. [30] that Al-Zr pairs form more easily than Ni-Zr pairs in random-packed Zr-Ni-Al supercooled liquid. Therefore, two kinds of strong pairs exist, i.e. Zr-Al and Zr-Ni, leading to a variety of local environments. A binary Al-Zr system can lead to many intermetallic compounds^39^. A study of the principal clusters indicates that the most stable forms are: Al_3_Zr, Al_2_Zr, Al_3_Zr_2_ and AlZr^39^. Even if these nanocrystalline phases form in our metallic glass during the annealing, they have low scattering power and are therefore not visible in the results shown in Figs 1 and 7. An exception may be the Al_3_Zr_2_ phase.

## Correlation between the atomic structure and strength

**Figure 9:**
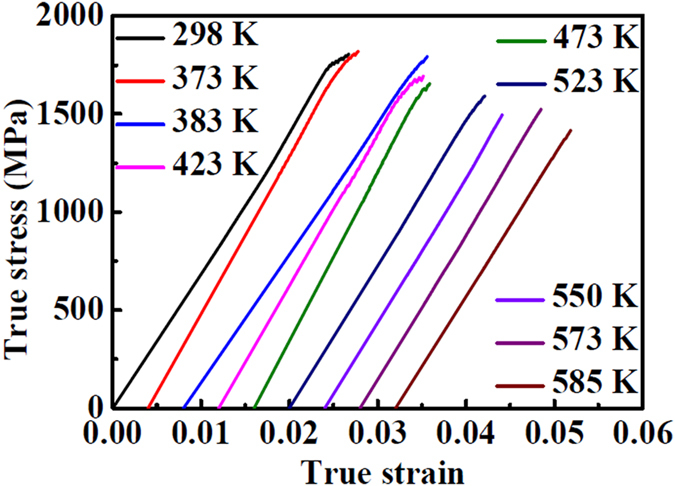
True stress-true strain curves of compressive tests at different temperatures.

Compression tests at different temperatures were conducted to obtain the yield strength at different temperatures (Fig. 9 and Table 3), revealing a decreasing trend with increasing temperature. From the true stress-true strain curves, the samples from 298 K to 473 K show a gradually decreasing ductility. Above 473 K, embrittlement occurs. The annealing of metallic glass at relatively low temperatures below the glass transition temperature is found to induce its embrittlement or a large reduction of toughness^44^,^45^,^46^.

In conventional understanding of the ductile-brittle transition (DBT) of engineering alloys the low temperature brittleness is replaced by ductile behavior at elevated temperature^47^,^48^. However, an opposite trend is found in our results (see Fig. 9), that is the samples become brittle after annealing and testing at high temperature, whilst showing some ductility in the as-cast condition. This phenomenon has been reported previously, for example by Li *et al*.^46^ and by Pan *et al*.^49^. The latter authors summarized the mechanism of the DBT as a change from propagation of multiple shear bands and corresponding ductility at low temperature to single shear band and brittleness at high temperature. They attribute this to temperature dependence of shear transformation zone (STZ) volumes.

A better interpretation for the thermal annealing-induced embrittlement is the reduction of the deformation flow units such as free volume after annealing^50^. Through the movement of flow units, metallic glass can exhibit plastic flow, during which the flow units can be activated easily in some local atomic sites with large density of free volume and low flow resistance^51^,^52^,^53^, which can lead to profuse shear bands^50^. The density of free volume reaches a critical value when the external stress approaches the yield strength of the metallic glass^54^.

Upon annealing at relatively low temperatures, such as below 523 K (Fig. 9), there is no major change of the microstructure of the glass. In our tests, annealing time and temperature changes cause some different microstructures at different temperatures. Flow units as a kind of intrinsically heterogeneous soft zone are embedded in a hard matrix. Shear bands or cracks propagate in the hard matrix more quickly than in the soft flow units. For the as cast sample at room temperature (298 K), a relatively large amount of flow units is distributed in the matrix. Thus, plastic flow can be observed because of the blunting at the crack tip by flow units. However, with the annealing time and temperature increasing, the amount of flow units is significantly reduced. Then, many crack tips cannot be blunted by the flow units, resulting in a decrease of the true strain, such as from 373 K to 523 K in Fig. 9. With more time and higher temperature annealing, the amount of flow units decreases further, and shear bands or cracks will propagate through the entire sample which can result in nearly-zero macro-plasticity^46^, such as at 550 K, 573 K and 585 K in Fig. 9.

The view of the heterogeneous structure has gradually emerged from a large number of studies, such as non-exponential relaxation phenomena in glass formers^54^,^55^,^56^, modelling of density heterogeneity^57^ and mechanical behavior of metallic glasses^46^.

**Figure 10:**
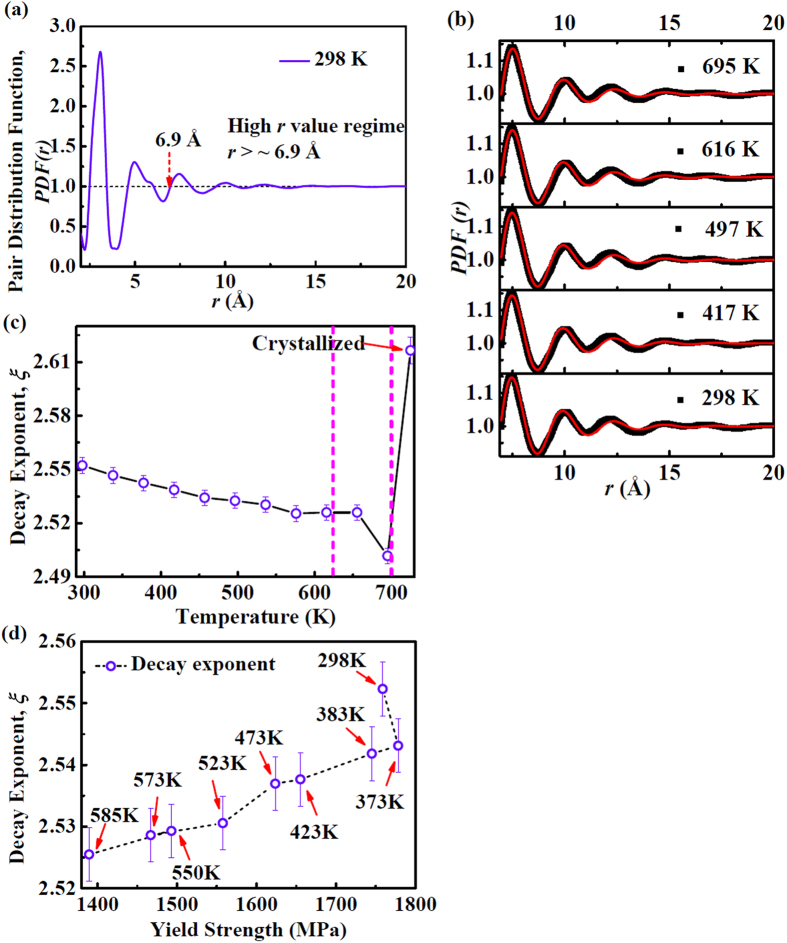
Correlation between the first diffraction shell and medium range order. (**a**)The pair distribution function, *PDF*(*r*) at 298 K. The dotted arrow indicates the onset of the third shell in figure (**a**) and *r* = 6.9 Å. (**b**) *PDF*(*r*) fitted by a power-law function (equation 2) accompanied with an exponential decay at 298 K, 417 K, 497 K, 616 K and 695 K, respectively. (**c**) The fitting decay exponent, *ξ*, as a function of temperature. (**d**) The decay exponent, *ξ*, as a function of yield strength at different temperatures.

To explore the relationship between the properties and the atomic structure evolution with temperature, generally, the structure in the medium range is correlated with the macroscopic properties in metallic glasses. The pair distribution function of metallic glasses in the high *r* value regime can be fitted by a power-law function with an exponential decay as shown in Fig. 10(a) and (b)^23^:


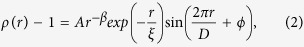


where *A* is a normalization constant, *β* is a scaling exponent (0.69), *ξ* is a decay exponent, *D* is the oscillation frequency, and *Φ* is the phase shift constant^23^. The first two peaks are omitted as corresponding to short-range order, and the choice of the starting value of *r* = 6.9 Å for fitting equation (2) is made by noting that *PDF* goes through value of 1 at that distance. The medium-range order of metallic glasses has been found to be predicted by Eq. (2).

To extract information on the medium-range order, the *PDF*(*r*) in the high *r* value regime is fitted by Eq. (2), and the fitting curves at different temperatures are plotted in Fig. 10(b). The best fitting parameters were found to be: *A* = 11.11 ± 0.82, *β* = 0.69, *D* = 2.434 ± 0.004. The decay exponent, *ξ*, estimated from the fitting process exhibits a slowly decreasing trend versus temperature increasing before the glass transition, as shown in Fig. 10(c). In the supercooled liquid region, the decay exponent decreases rapidly with increasing the temperature from 656 K to 695 K. The *ξ* parameter is associated with the yield strength of metallic glasses^14^. Furthermore, the yield strength has a close relationship with the glass transition temperature^58^. From Fig. 10(d), the decay exponent, *ξ*, decreases with the yield strengths decreasing from room temperature to 585 K.

The exponent, *ξ*, in Eq. (2) as the correlation length of MRO of glassy phase provides a fingerprint reflecting the correlation of the atoms in elementary deformation units. A large decay exponent suggests that the correlation between atoms is weak^59^. With increasing temperature, the decreased decay exponent suggests a strong correlation between atoms.

**Figure 11:**
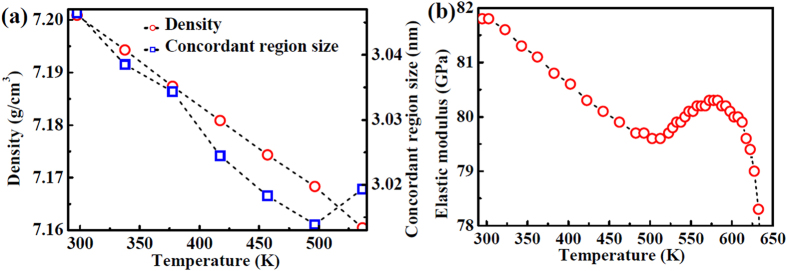
(**a**) The density and the concordant region size calculated by *ρ* = *M*/*A*_*v*_ · *V*_*a*_, where *A*_*v*_ is Avogadro constant, *M* is the molar weight, and *V*_*a*_ is the atomic volume from 
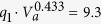
 (*q*_*1*_ is the position of first maximum in structure factor)^25^. (**b**) The measured elastic modulus with increasing temperature.

The deformation unit operation includes a group of atoms concordantly shifting and forming a concordant region^14^. The stress increase can bring out an expansion of this concordant region due to more atoms participating in rearrangement^14^. The formation of the concordant deformation region is actually a response of the metallic glass to the external load, which is manifested in a redistribution of the local stresses in an elastic medium^9^,^60^. This stress redistribution is carried out by an elastic interaction between atoms over a distance of shear wave propagation^16^. Thus, the concordant region size [in Fig. 11(a)], *d*, can be assumed to be *d* = *v*_*s*_*τ*, where *v*_*s*_ is the shear wave speed, and *τ* is the relaxation time that depends on temperature. In the present study, the *τ* value (approximately 1~2 ps) is a constant since the experiments were carried out at room temperature^16^. The shear wave speed can be calculated by *v*_*s*_ = (*G*/*ρ*)^1/2^, where *G* is the shear modulus, derived from independent elastic modulus measurement shown in Fig. 11(b) and the temperature variation of Poisson’s ratio taken from ref. [61], and *ρ* is density of the metallic glass shown in Fig. 11(a). Accordingly, it can be seen that the concordant region size is determined by the propagation of the shear wave^14^, which further indicates that the shear banding behavior is related to the shear wave propagation.

Thus, concordant shifting of atoms in the metallic glasses with large decay exponent, such as the Ni_62_Nb_38_^14^ and Zr_64.13_Cu_15.75_Ni_10.12_Al_10_, requires a higher activation energy. This corresponds to a high elastic modulus and a high yield strength, which is consistent with our above discussion that the metallic glass with high yield strength requires a large activation energy to form the concordant region.

## Conclusions

*In-situ* X-ray synchrotron diffraction is a well-suited technique to systematically explore the structural evolution of metallic glasses on the atomic level. The present work provides information on the atomic arrangement of the Zr_64.13_Cu_15.75_Ni_10.12_Al_10_ metallic glass heated to elevated temperatures in reciprocal space and in real space. In the range of 298 K to 699 K some structural changes occur involving rearrangements at the atomic level. The thermal expansion coefficient for individual coordination shells obtained from *PDF* can be summed to within experimental error of the macroscopic bulk coefficient. The atomic movement between the first and second coordination shell can account for the apparent contraction of the first shell on heating. Modulation of the local density provides sites conducive to formation of stable nuclei which allow subsequent nanocrystallisation. The evolution of the structure leads the yield strength of the metallic glass to decrease with increasing temperature, which is attributed to the formations and changes of flow units.

## Methods

Alloy ingots with a nominal composition of Zr_64.13_Cu_15.75_Ni_10.12_Al_10_ (at. %) were prepared by arc melting (at least four times remelting was employed to ensure chemical homogeneity) from pure metallic elements in a Ti-gettered argon atmosphere. The ingots were quenched into rod-like samples with 1 mm diameter × 20 mm length and 2 mm diameter × 70 mm length by copper mould casting. The as-cast metallic glass rods were cut into a size of 1 mm diameter × 2 mm length by a diamond saw for synchrotron radiation measurements. The two ends of the samples were carefully polished to be parallel. *In-situ* annealing experiments were carried out using high-energy X-ray synchrotron radiation at the P02.1 beamline of the PETRA III electron storage ring (DESY Hamburg, Germany). A quartz capillary with 1.2 mm diameter and 20 μm wall thickness served as a container for holding the samples. The temperature control was to within ±1 K^62^.The beam size was 0.6 × 0.6 mm^2^ and the wavelength was 0.20728 Å. The diffracted photons were collected with a 2D detector (Perkin-Elmer PE1621). Heating from room temperature up to crystallization was carried out at a heating rate of 5 K/min. The obtained two-dimensional X-ray diffraction patterns were integrated using the programme FIT2D^63^. To characterize the crystallization behaviour of the metallic glass, a NETZSCH DSC 204F1 differential scanning calorimeter (DSC) was used under a continuous nitrogen flow and a heating rate of 5 K/min. Thermal expansion measurements were carried out by a NETZSCH DIL402 dilatometer with a heating rate of 5 K/min from room temperature up to the glass transition temperature. The short cylinder samples were compressed by a MTS CMT 5205 mechanical testing machine at a constant strain rate of 2 × 10^−4^ s^−1^, and in a temperature range from room temperature to 585 K. The elastic modulus was measured by RFDA MF SYSTEM 21 from room temperature to 670 K.

## Additional Information

**How to cite this article**: Tong, X. *et al*. Structural evolution and strength change of a metallic glass at different temperatures. *Sci. Rep.* **6**, 30876; doi: 10.1038/srep30876 (2016).

## Figures and Tables

**Table 1:** The thermal expansion coefficients of different shells in *PDF* from 298 K to 616 K.

No.	Thermal expansion coefficient (K^−1^)	Standard error
The first shell (*r*_*1*_)	−1.35 × 10^−5^	0.08 × 10^−5^
The second shell (*r*_*2*_)	1.24 × 10^−5^	0.32 × 10^−5^
The third shell (*r*_*3*_)	0.86 × 10^−5^	0.24 × 10^−5^
The fourth shell (*r*_*4*_)	0.36 × 10^−5^	0.10 × 10^−5^
Total	1.11 × 10^−5^	

**Table 2:** Ten possible atomic pairs in the Zr_64.13_Cu_15.75_Ni_10.12_Al_10_ and theoretical bond length of these atomic pairs and their probabilities and weighting factors calculated at *q* = 0 Å^−1^.

Atomic pair	Bond length (Å)	Probability, *P*_*ij*_	Weighting factor, *W*_*ij*_
Zr-Zr	3.20	0.4113	0.5576
Zr-Cu	2.88	0.2020	0.1986
Zr-Ni	2.85	0.1298	0.1232
Zr-Al	3.03	0.1283	0.0565
Cu-Cu	2.56	0.0248	0.0177
Cu-Ni	2.53	0.0319	0.0219
Cu-Al	2.71	0.0315	0.0101
Ni-Ni	2.50	0.0102	0.0068
Ni-Al	2.68	0.0202	0.0062
Al-Al	2.86	0.0100	0.0014

**Table 3:** Yield strengths of the metallic glass in compression tests at different temperatures.

**Temperature (K)**	298	373	383	423	473	523	550	573	585
**Yield Strength (MPa)**	1759	1778	1745	1655	1624	1558	1467	1493	1390
